# Prediction for Distant Metastasis of Breast Cancer Using Dynamic Contrast-Enhanced Magnetic Resonance Imaging Images under Deep Learning

**DOI:** 10.1155/2022/6126061

**Published:** 2022-06-08

**Authors:** Li Li, Hongzhe Tian, Baorong Zhang, Weijun Wang, Bo Li

**Affiliations:** Department of Medical Iconography, Baoji Central Hospital, Baoji 721008, Shaanxi, China

## Abstract

This research aimed to explore the effect of using magnetic resonance imaging (MRI) radiomic features to establish a model for predicting distant metastasis under dynamic contrast-enhanced MRI imaging with deep learning algorithms. The deep learning algorithm was used to segment the images. A total of 96 cases with 100 lesions were included in the metastatic group, including 2 cases of bifocal breast cancer and 2 cases of multifocal breast cancer. There were 192 cases in the nonmetastatic group, with 197 lesions, including 5 cases of multifocal breast cancer. After dynamic contrast-enhancement, the morphological features and grayscale statistical features were extracted from the lesions to establish a prediction model through sum-sum check and feature dimension reduction. The accuracy, sensitivity, specificity, and area under receiver operator characteristic curve (AUC) of prediction models based only on imaging features were compared with those created by combining radiomic features with clinical and pathological features. The created predictive model based on radiomic features for distant metastases in breast cancer showed a sensitivity of 66.7%, a specificity of 84.2%, an accuracy of 78.3%, and an AUC of 0.744. The sensitivity of the prediction model for distant metastasis of breast cancer was 67.7%, the specificity was 86.8%, the accuracy was 80.5%, and the AUC was 0.763. Bone, lung, and liver were the most common distant metastatic sites of breast cancer. Under the dynamic contrast-enhanced MRI of deep learning, the prediction model combining radiomic features with clinical and pathological features showed better predictive performance.

## 1. Introduction

Breast cancer is one of the most common female malignancies in the world. If distant metastasis occurs, it is clinically stage IV and the prognosis is not good [1]. Bone, lung, and liver are the most common distant sites of breast cancer. Studies have shown that the median survival time of patients with liver metastases is 4–22 months and palliative systemic therapy is usually the treatment. The median survival time for patients with only bone metastases and no other organ metastases is 45 months. The median survival of patients with lung metastases is 22 months. However, the median survival time for patients who do not receive treatment is 1.2 months. Therefore, early detection of breast cancer with distant metastasis can develop individualized treatment plans, which have specific significance for predicting the survival time of patients and improving the prognosis of patients [2]. Tumor heterogeneity is one of the characteristics of malignant tumors, which can be divided into spatial heterogeneity and temporal heterogeneity [3]. Tumor heterogeneity also maps the properties of various regions within the tumor, and tumors with high heterogeneity have poorer prognosis than those of with low heterogeneity [4]. Pathological examination is the gold standard for breast cancer diagnosis. However, thick needle biopsy and postoperative pathological diagnosis have certain limitations; that is, the above-mentioned examinations are invasive and can only provide information on part of the tumor but cannot reflect information on the entire tumor [5]. Therefore, it is necessary to find a noninvasive assay to map the heterogeneity within the tumor. Imaging examinations can provide a more detailed visualization of the tumor and can monitor disease progression and treatment during repeated treatment, to some extent complement the limitations of pathological examinations. In addition, imaging examinations also have a strong potential to guide treatment [6]. Breast magnetic resonance imaging (MRI) can comprehensively evaluate the overall information of the tumor by observing the morphological and hemodynamic characteristics of the lesions, reflecting the heterogeneity of the tumor. As the most sensitive imaging method for breast cancer, MRI has been widely used in clinical practice [7, 8]. The main processes of radiomics analysis are as follows: First, high-quality images are obtained, and then manual or automatic image segmentation is performed on the tumor by delineating a region of interest (ROI), which may include all or part of the tumor. After the image segmentation is completed, the segmented area can be formed into a three-dimensional image, and the volume can be obtained. A large amount of internal quantitative information is extracted through the software and combined with the corresponding clinical information, genetic information, serum markers, and histological information to make corresponding predictions [3, 9]. However, these studies have certain limitations because patient outcomes are not determined by a single prognostic factor.

Artificial intelligence (AI) has been developed to automatically quantify data features in medical images. Deep learning is a subset of artificial intelligence that can automatically learn features from sample images to match the performance of the human brain in specific task applications. Furthermore, many studies have demonstrated that the expressive power of deep learning is particularly promising [10]. Automated capabilities of AI have the potential to enhance clinician expertise, including accurate volumetric description of tumor size over time, parallel tracking of multiple lesions, the impact of intratumoral phenotypic differences, and the ability to compare the individual tumors to comparable cases with unlimited potential database for cross-linked prediction results [11]. AI shows the potential to significantly enhance the efficiency, reproducibility, and quality of tumor measurements through automated segmentation. With the rapid expansion of computing speed and the improvement of the efficiency of AI algorithms, the analysis of cancer lesions in the future may no longer require a separate segmentation step, and the whole body imaging data can be directly evaluated by AI algorithms [12]. The systematic approach can also analyze organs for structural abnormalities, possibly pathological, that cannot be detected by human vision. In terms of imaging data, the benign and malignant diagnosis of suspected lesions will ultimately be interpreted intuitively by imaging experts. The clinician applies the experience and expertise of physicians to deal with similar problems using subjective and qualitative characteristics [13]. Computer aided diagnosis (CADx) systems use systematic interventions that quantify tumor characteristics, enabling the use of more reproducible descriptors. The CADx system has been used for the diagnosis of thin-slice CT pulmonary nodules and multiparametric MRI [14] prostate lesions. Especially in the case of different interpretations by doctors, the results of AI have greater auxiliary reference value. The objective of this work was to explore the predictive effect of a dynamic contrast-enhanced MRI prediction model based on the fully connected structure of the deep matrix decomposition algorithm for distant metastasis of breast cancer.

## 2. Materials and Methods

### 2.1. Research Objects

Patients who underwent breast MRI in hospitals from January 2018 to November 2020, were included in this research. This research was approved by the Ethics Committee of the Hospital, and the families of the patients included signed the informed consent. Inclusion criteria were defined as follows: I. Patients with complete breast MRI image data and patients whose image quality could meet the diagnostic criteria; II. Patients with invasive cancer, which was confirmed by surgical pathology; III. Patients who were treated according to breast cancer diagnosis and treatment standards before and after surgery received conventional treatment. Patients performed with chest CT, abdominal and pelvic ultrasound, and bone scan were followed up, and brain MRI was performed for those with cerebral symptoms. 96 patients with distant metastasis were identified as the metastatic group. According to statistical rules, 192 patients without distant metastasis were randomly selected from the patients who met the inclusion criteria as the nonmetastatic group. Exclusion criteria were: ① patients with other diseases such as traumatic brain injury; ② patients with serious mental illness who can not cooperate with the study.

### 2.2. Equipment and Methods

Two types of MRI machines (1.5 T MRI machine and 3.0 T MRI machine) were used for scanning. An 8-channel phased surface coil was selected for breast examination. The patient was in the prone position, and the breasts were naturally sagging inside the coil. The nonenhanced scans were performed using cross-sectional FSE t2 WI sequences (time of repetition (TR) was 625 ms and time of echo (TE) was 12 ms) and fat-suppressed t3 WI sequences (TR was 6345 ms and TE was 67 ms). The layer thickness was 4.6 mm, the layer spacing was 0.6 mm, the matrix was 385 × 232, and the number of excitation (NEX) was 2. For the breast volume imaging (VIBRANT), the TR was 4.3 ms, the TE was 2.8 ms, the rotation angle was 16°, and then the optimized parallel acquisition 3D fast gradient echo sequence was adopted. Matrix was 255 × 126, the field of view (FOV) was 28 cm × 27 cm, the layer thickness was 1.7 mm, and NEX was 2. Before the injection of the contrast agent, the mask was scanned firstly, and then the contrast agent GD-DTPA was injected through the dorsal vein with a high-pressure syringe group infusion at a dose of 0.3 mL/kg and a rate of 2.3 mL/s. An equal volume of normal saline was then injected immediately, followed by a delay of 9 minutes, with a single scan time of 62–102 seconds. Finally, the time-lapse transaxial volume imaging sequence was scanned.

### 2.3. Deep Learning Algorithms to Segment Images

**Figure 1:**
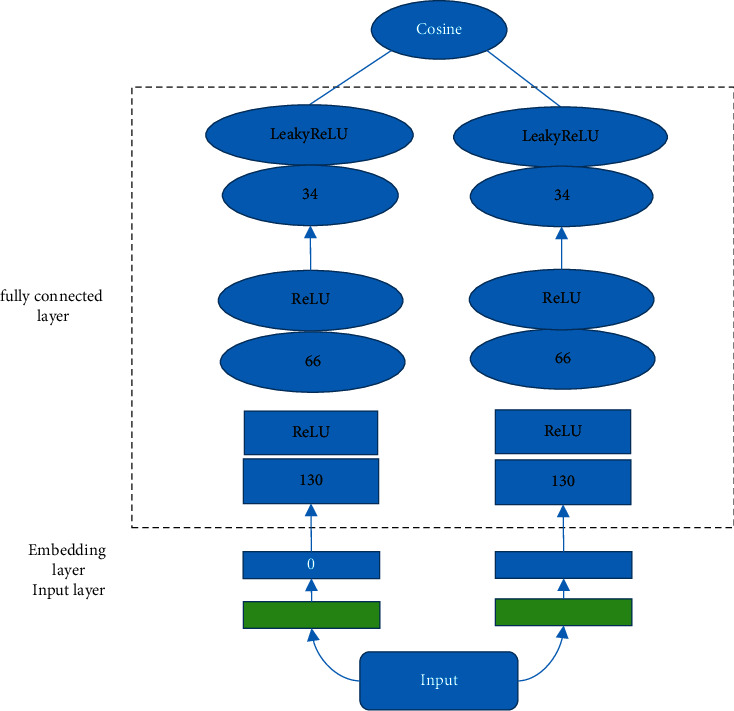
The deep matrix model of the fully connected structure.

The deep matrix factorization model using the fully connected structure mainly includes input layer, embedding layer, fully connected layer, and output layer, as shown in Figure 1. In the input layer, the preprocessed pathological information and image feature matrix *X* of size *M* × *N* are used as the input of the network, and the pathological information *X*_*ij*_. The left input is *X*_*i*_ and the right input is *X*_*j*_, corresponding to the *ith* row and *jth* column of matrix *X*, respectively. In the embedding layer, the dimension of the latent eigenvector of the input matrix is set to *k*, *C*_*j*_, and *C*_*a*_ are the latent eigenvectors of the row and column obtained by nonlinear transformation; *S*_*j*_ and *S*_*a*_ are the eigen projection matrix of the matrix row vector and column vector, respectively, and *µ* (C) is the nonlinear activation function.

The depth matrix decomposition model of the fully connected structure mainly includes input layer, embedding layer, fully connected layer, and output layer. In the input layer, the preprocessed pathological information and image feature matrix C are used as the input of the network. Pathological information is C_*lg*_, observation value C_*lg*_ the left input C_*lg*_ and right input *C*_*lg*_ correspond to the L-th row and *g*-th column of matrix *C*, respectively. In the embedding layer, if the dimension of the latent eigenvectors of the input matrix is *C*_*j*_ and *C*_*a*_, then they are the latent eigenvectors of rows and columns obtained by nonlinear transformation, respectively. *S*_*j*_ and *S*_*a*_ are the feature projection matrices of the matrix row vector and column vector, respectively, and µ(C) is the nonlinear activation function:

(1) $$\begin{array}{c} {\text{C}}_{j}=µ\left(C^{l}S_{j}\right),\\ {\text{C}}_{a}=µ\left(S_{a}C_{jG}\right). \end{array}$$

In the fully connected layer, the first and second fully connected layers used the ReLU function as the activation function of neurons, and the third fully connected layer took the Leaky ReLU function as the activation function. If the output of the multilayer perceptron of this line vector and column vector was denoted by *D* and *R*, respectively, then *B* was a fixed parameter in the interval 1 to +∞. The expressions for ReLU and Leaky ReLU are expressed as the following equations:

(2) $$\begin{array}{c} \text{ReLU: }v=\left\{\begin{array}{cc} c, & \text{if }x⩾0\\ 0, & \text{if }x\langle )0 \end{array}\right.,\\ \text{LeakyRelu: }v=\left\{\begin{array}{cc} c, & \text{if }x⩾0\\ \frac{c}{b}, & \text{if }x\langle )0 \end{array}\right). \end{array}$$

The output layer and loss function of the model were populated with clinicopathological information through a deep matrix factorization network. This was a supervised training process, so the cross-entropy was undertaken as the loss function. The cross-entropy loss function can be expressed as the following equation:

(3) $$\begin{array}{c} L=-\sum_{\left(l,g\right)\in Y}V_{\mathrm{lg}}\text{log}V_{\mathrm{lg}}^{'}+\left(2-V_{\text{lg}}\right)\text{log}\left(2-V_{\mathrm{lg}}^{'}\right). \end{array}$$

### 2.4. Feature Dimension Reduction

The purpose of feature dimension reduction was to reduce the redundancy of the original feature data and present as much original information as possible using the fewest features. The rank-sum test was initially performed on the 341 radiomic features extracted from the two groups, and the features with *p* > 0.001 were excluded for preliminary screening. The least absolute contraction and selection operator (LASSO) algorithm was used to select 87 features with *p* ≤ 0.001. It mainly reduced some unimportant variables to none by screening, so as to complete variable selection and parameter estimation at one time, thereby reducing the amount of calculation and completing the process of rapid variable selection. In this work, feature dimension reduction was performed on the first stage lesion image dataset after dynamic contrast enhancement.

### 2.5. Establishment and Performance Evaluation of the Classification Model

Based on the radiomic features of metastatic group and nonmetastatic group lesions in the later stage of dynamic contrast-enhancement, a prediction model was established combined with the radiomic features of lesions in the later stage of dynamic contrast-enhancement and clinical and pathological features. The feature parameter set obtained after feature dimension reduction was input into the ensemble learning classifier for classification. The classification sensitivity, specificity, accuracy, and area under the receiver operating characteristic curve (AUC) of the model were calculated using ten-fold cross-validation. The average values were undertaken as the final prediction results to compare the prediction effect of the model.

### 2.6. Statistical Analysis

A univariate analysis was performed for clinical and pathological features. The Kolmogorov–Smirnov test was selected for a continuous variable to see if it follows a normal distribution. The *t*-test was used for normal distribution, and the Kruskal–Wallis *U* test was used for statistical analysis for skewed distribution. Statistical analysis of discrete variables was performed using the *χ*^2^ test or Fisher's test. *p* < 0.05 meant the difference was statistically significant.

## 3. Results

### 3.1. Clinical Features

The 96 cases (6 deaths) in the metastatic group were all female, aged 20–72 years old, with 100 lesions, including 2 cases of bifocal breast cancer and 2 cases of multifocal breast cancer. There were 192 cases in the nonmetastatic group, all women aged 26–77 years old, with 197 lesions, including 5 cases of multifocal breast cancer. There was no significant difference in age between the two groups by the Mann–Whitney *U* test (*p* < 0.05). As shown in Table 1, there were 88 cases of nonspecific invasive ductal carcinoma, 5 cases of invasive micropapillary carcinoma, 2 cases of invasive lobular carcinoma, 3 cases of mucinous carcinoma, and 2 cases of invasive papillary carcinoma in the metastatic group. The nonmetastatic group included 184 nonspecific invasive ductal carcinomas, 2 invasive micropapillary carcinomas, 2 mucinous carcinomas, 5 invasive cribriform carcinomas, 2 invasive ductal carcinomas with squamous cell differentiation, and 2 tubular carcinomas. In the metastatic group, 55 (55%) were masses and 45 (54%) were nonmass. In the nonmetastatic group, 159 (80.7%) had mass lesions and 38 (19.3%) had no mass lesions, showing no significant difference between the two groups (*p* < 0.05).

### 3.2. Evaluation of Segmentation Effect of Dynamic Contrast-Enhanced MRI Imaging Based on Deep Learning

**Figure 2:**
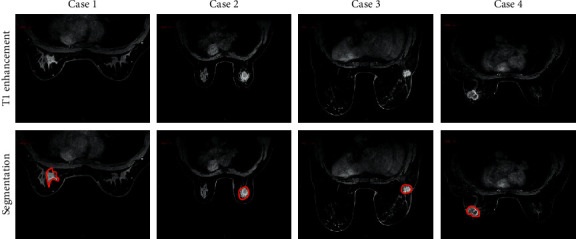
MRI images of breast cancer distant metastasis and the result of algorithm segmentation of the MRI image target area. Case 1 was 58 years old, case 2 was 35 years old, case 3 was 49 years old, and case 4 was 62 years old. The red box marked the specific observation positions.

The learning of the diagnostic network was monitored using pathological findings, i.e., whether a lesion in MRI was benign or malignant, as a ground truth. The 3D network was implemented using Python and TensorFlow deep learning framework under the Ubuntu 17.01 system using CUDA 8.2, cuDNN 6.1, and the TITAN Xp GPU server. The model was trained from scratch with randomly initialized weights and Gaussian distribution (*μ* = 0 and *σ* = 0.01). During stochastic gradient descent, an adaptive moment estimation optimizer was used to update the parameters. The patch size was set to 338 × 218 × 125 and the batch size was set to 13. It took 30 minutes to train one epoch on one graphics processing unit (GPU), and the model was fused after about 89 epochs.  Figure 2 showed the T1-enhanced MRI images of 4 patients. After algorithm segmentation, the lesions can be clearly located, which was convenient for doctors to diagnose the patient's condition.

### 3.3. Examination Results

Among the 100 breast cancer lesions of 96 patients in the metastatic group, 16 (16%) were luminal A type, 49 (49%) were luminalB type, 16 (16%) were Her-2 overexpression type, 17 were triple-negative type (17%), and 2 (2.0%) were no molecular typing (incomplete immunohistochemical results). Among the 197 breast cancer lesions of 192 patients in the nonmetastatic group, 25 (12.7%) were luminalA type, 134 (68.0%) were luminalB type, 13 (6.6%) were Her-2 overexpression type, and 25 (12.7%) were triple-negative type. There was a statistically significant difference in molecular typing between the two groups of (*p* < 0.05). The characteristics of different molecular types of metastatic group breast cancer distant metastasis are shown in Table 2.

The morphological features, grayscale statistical features, texture features, and wavelet features were extracted from 297 breast cancer lesions. After inspection and feature dimensionality reduction, images were finally selected for statistics. It was found that significant radiomic features included 4 morphological features, 3 grayscale statistical features, 9 texture features, and 16 wavelet features, which were then used to establish prediction models. Univariate clinical and pathological features analysis showed that there were significant differences in lactation history, abortion history, birth history, birth times, menstrual history, lymph node metastasis, ER expression, PR expression, HER-2 expression, and molecular typing between the two groups (*p* < 0.05).

**Figure 3:**
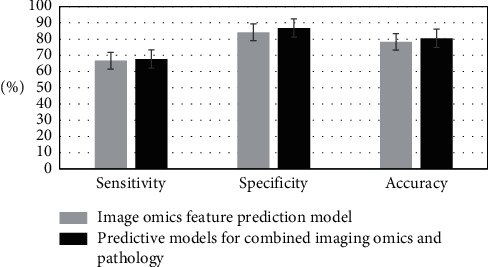
Comparison of prediction model based on radiomic features and prediction model combining with radiomic and pathology.

As shown in Figure 3, the breast cancer distant metastasis prediction model created with radiomic features showed a sensitivity of 66.7%, a specificity of 84.2%, an accuracy of 78.3%, and an AUC of 0.744. The breast cancer distant metastasis prediction model established by the combination of radiomic features and clinical and pathological features showed a sensitivity of 67.7%, a specificity of 86.8%, an accuracy of 80.5%, and an AUC of 0.763. There were statistically significant differences in sensitivity, specificity, and accuracy between the predictive model of radiological features and that combined with radiopathology (*p* < 0.05).

## 4. Discussion

Breast cancer is a highly heterogeneous tumor, and the main cause of death in breast cancer patients is generally not the primary tumor, but distant metastasis [15]. Over the past few decades, the incidence of distant metastasis and death in breast cancer patients has decreased significantly due to advances in surgical methods and systematic neoadjuvant chemotherapy [16]. However, distant metastasis occurs in about 19%–29% of patients regardless of the treatment regimen. Most distant metastasis patients have poor prognoses, and early detection of distant metastasis patients is beneficial to predict their survival time, and individualized treatment plans can be formulated for different individuals [17]. Previous prediction models for distant metastasis of breast cancer usually have clinical and pathological indicators, but imaging-based prediction models for distant metastasis are rarely reported [18]. MRI has been widely used in the preoperative assessment of breast cancer lesion size and extent. Undetected lesions can also be found in ipsilateral and contralateral breast lesions, and it is an important supplement to preoperative mammography and ultrasound [19, 20]. At present, radiologists still diagnose breast MRI images mainly by observing pathological features with the naked eye. However, the information obtained from the pathological changes observed by the human eye is very limited, and how to extract more features that cannot be observed by the naked eye has become a new challenge in imaging [21]. In previous decades, oncologists and radiologists have been interested in the clinical application of quantitative features of images because of major advances in the field of medical imaging, resulting in radiomics [22]. Using radiomics, physicians can extract rich quantitative features from digitized medical images, followed by high-throughput output. These quantitative features include tumor size, shape, signal intensity, and texture features, which provide comprehensive information for revealing tumor characteristics [23].

The common Luminal bone metastases and common triple-negative lung metastases found in this work are consistent with those reported in the literature, while the Her-2-overexpressing liver metastases are inconsistent with literature reports, which may be related to the few distant metastasis cases included in this study [11, 24]. In this work, dCE-MRI dynamic contrast-enhancement phase I images of 96 patients in the metastatic group (100 lesions) and 192 patients in the nonmetastatic group (197 lesions) were collected. The ROI map of the lesion layer was drawn, and 35 imaging characteristic parameters of the lesion were collected to create a prediction model for breast cancer patients with distant metastasis in this work. The results revealed that the sensitivity, specificity, accuracy, and AUC of this model were 66.7%, 84.2%, 78.3%, and 0.744, respectively, showing a good prediction effect. In addition to collecting the dCE-MRI dynamic contrast-enhancement postphase images in the metastatic group and nonmetastatic group, the clinical-pathological features of the above cases were collected, and a joint prediction model of breast cancer distant metastasis was established by combining with the radiomic features. The model showed a sensitivity of 67.7%, a specificity of 86.8%, an accuracy of 80.5%, and an AUC of 0.763. The above results were higher than prediction models based solely on radiomic features, implying that radiomic features combined with clinical and disease physiological features can better predict distant metastasis in breast cancer patients.

## 5. Conclusion

Under the dynamic contrast-enhanced MRI of deep learning, both prediction models using only radiomic features parameters and radiomic features combined with clinical and pathological features can achieve good prediction performance. However, the prediction model of radiomic features combined with clinical and pathological features showed better predictive performance. The prediction model established by radiomic features combined with clinical and pathological features showed better predictive performance for breast cancer patients with distant metastasis, which can provide certain reference value for the formulation of treatment plans. There were also many deficiencies in this work. The sample size was too small, and more experimental people should be included. Clinical trials should not be conducted in a single area or a small area but should be conducted in a multicenter and large-sample hospital.

## Figures and Tables

**Table 1:** Clinical characteristics of the patients.

	Metastatic group (cases)	Nonmetastatic group (cases)
Nonspecific invasive ductal carcinoma	88	184
Invasive micropapillary carcinoma	5	2
Mucinous carcinoma	3	2
Invasive lobular carcinoma	2	0
Invasive papillary carcinoma	2	0
Invasive cribriform carcinoma	0	5
Invasive ductal carcinoma with squamous cell differentiation	0	2
Tubular carcinoma	0	2

**Table 2:** Breast cancer distant metastasis with different molecular types (%).

	Bone	Lung and pleura	Liver	Brain	Other part
Luminal A type (16 cases)	9 (56.3)	3 (18.8)	3 (18.8)	5 (31.3)	2 (12.5)
Luminal B type (49 cases)	37 (75.6)	20 (40.8)	16 (32.7)	8 (16.3)	1 (2.04)
Her-2 overexpression type (16 cases)	4 (25)	7 (43.8)	10 (62.5)	2 (12.5)	1
Triple-negative type (17 cases)	8 (47.1)	8 (47.1)	5 (29.4)	4 (23.5)	1 (5.9)

## Data Availability

The data used to support the findings of this study are available from the corresponding author upon request.
